# Novel Temperate Phages of *Salmonella enterica* subsp. *salamae* and subsp. *diarizonae* and Their Activity against Pathogenic *S*. *enterica* subsp. *enterica* Isolates

**DOI:** 10.1371/journal.pone.0170734

**Published:** 2017-01-24

**Authors:** Lenka Mikalová, Juraj Bosák, Hana Hříbková, Daniela Dědičová, Oldřich Benada, Jan Šmarda, David Šmajs

**Affiliations:** 1 Department of Biology, Faculty of Medicine, Masaryk University, Kamenice 5, Brno, Czech Republic; 2 National Reference Laboratory for Salmonella, The National Institute of Public Health, Šrobárova, Prague, Czech Republic; 3 Institute of Microbiology of ASCR, v.v.i., Vídeňská, Prague, Czech Republic; Centro Nacional de Biotecnologia, SPAIN

## Abstract

Forty strains of *Salmonella enterica* (*S*. *enterica*) subspecies *salamae* (II), *arizonae* (IIIa), *diarizonae* (IIIb), and *houtenae* (IV) were isolated from human or environmental samples and tested for bacteriophage production. Production of bacteriophages was observed in 15 *S*. *enterica* strains (37.5%) belonging to either the subspecies *salamae* (8 strains) or *diarizonae* (7 strains). Activity of phages was tested against 52 pathogenic *S*. *enterica* subsp. *enterica* isolates and showed that phages produced by subsp. *salamae* had broader activity against pathogenic salmonellae compared to phages from the subsp. *diarizonae*. All 15 phages were analyzed using PCR amplification of phage-specific regions and 9 different amplification profiles were identified. Five phages (SEN1, SEN4, SEN5, SEN22, and SEN34) were completely sequenced and classified as temperate phages. Phages SEN4 and SEN5 were genetically identical, thus representing a single phage type (i.e. SEN4/5). SEN1 and SEN4/5 fit into the group of P2-like phages, while the SEN22 phage showed sequence relatedness to P22-like phages. Interestingly, while phage SEN34 was genetically distantly related to Lambda-like phages (*Siphoviridae*), it had the morphology of the *Myoviridae* family. Based on sequence analysis and electron microscopy, phages SEN1 and SEN4/5 were members of the *Myoviridae* family and phage SEN22 belonged to the *Podoviridae* family.

## Introduction

At present, the *Salmonella* genus contains two species: *S*. *enterica* and *S*. *bongori* (V). *S*. *enterica* is further subdivided into six subspecies: *enterica* (I), *salamae* (II), *arizonae* (IIIa), *diarizonae* (IIIb), *houtenae* (IV), and *indica* (VI) [1–3]. Salmonelloses (infections transferred between humans and animals, mainly by contaminated food) represent an important, global, public health problem, with *S*. *enterica* subsp. *enterica* causing about 99% of the human cases [3–6]. Out of more than 2,600 *S*. *enterica* subsp. *enterica* serovars [7], relatively few serovars are important causative agents of salmonelloses [3]. While *S*. *enterica* subsp. *enterica* is typically found in warm-blooded animals, *S*. *enterica* subsp. *arizonae* and *S*. *bongori* are associated with cold-blooded animals. The other four subspecies of *S*. *enterica* can be isolated from both host types and can occasionally cause human infections [8–13].

Bacteriophages are the most abundant group of biological agents in our environment [14]. Tailed phages represent the dominant morphotype among characterized bacterial viruses as shown through electron microscopic examination of more than 5500 phages, in which 96% of phages were tailed [15]. Tailed phages also dominate with regard to the number of complete genome sequences; analysis of 607 complete phage genomes in the GenBank database revealed 506 (80%) tailed phages [16]. Tailed phages are in the *Caudovirales* order and are subdivided according to tail morphology into 3 families–*Myoviridae* (phages with long, contractile tails), *Siphoviridae* (phages with long, non-contractile tails), and *Podoviridae* (phages with short tails).

Based on a comparison of genome sequences of tailed enterobacterial phages [17], 77 *Salmonella* phages were found in 23 clusters including lytic phages (e.g. T4-like, T7-like, and SETP3-like) and temperate phages (e.g. Lambda-like, P2-like, and P22-like). In addition, 9371 prophages from 3298 *Salmonella* genomes were recently identified; out of them, 744 prophages belonged to the P22-like group and 4758 to the P2-like group [18].

*Salmonella* phages or prophages have been predominantly studied in strains of *S*. *enterica* subsp. *enterica*. In this communication, we focused on phage production by strains in the less common *S*. *enterica* subspecies *salamae*, *arizonae*, *diarizonae*, and *houtenae*. Genetic relatedness of identified phages and their activity spectra were determined. In addition, phages SEN1, SEN4/5, SEN22, and SEN34 were characterized by electron microscopy and whole genome sequencing.

## Materials and Methods

### Bacterial strains and culture media

From 1999–2005, 40 non-pathogenic strains of *S*. *enterica* subspecies *salamae*, *arizonae*, *diarizonae*, and *houtenae* (referred as Sen 1–40) were collected in the Czech Republic from human clinical samples and from environmental samples. All *S*. *enterica* strains were provided by The National Reference Laboratory for Salmonella, National Institute of Public Health (NIPH), Prague, Czech Republic. *Salmonella* strains were serotyped using the White-Kauffmann-Le Minor classification scheme [19] and their serotype characteristics are listed in S1 Table.

For cultivation of bacteria in liquid culture, tryptone yeast (TY) medium with the following composition was used: yeast extract (HiMedia, Mumbai, India) 5g/L, casein enzyme hydrolysate (HiMedia) 8g/L, and NaCl (Penta, Prague, Czech Republic) 5g/L. TY agar plates were supplemented with a 1.5% (w/v) of agar (Hi-Media).

### Identification of lysogenic strains

Forty *S*. *enterica* strains were tested for bacteriophage production using a cross test method where each strain was tested as a possible producer as well as a possible indicator strain. In addition, four standard *E*. *coli* indicators (K12-Row, C6 (φ), B1, and P400), which were available in our laboratory strain collection [20–21], were used to detect phage producers. Briefly, each *Salmonella* strain was inoculated from a fresh TY broth culture into an agar base layer, 1.5% (w/v), using a sterile needle and then cultivated at 37°C for 48 hr. The resulting macrocolony was killed using chloroform vapors (30 min) and the plate was overlaid with a top layer of 0.7% (w/v) agar enriched with a suspension of 10^7^ indicator bacteria. Phage production was evaluated after overnight cultivation at 37°C.

### Determination of phage titer and inducibility of phages

Phage producers were inoculated into TY broth and incubated overnight. The fresh culture was diluted hundredfold with TY broth and cultivated for an additional 7 hr (37°C, 200 rpm). Subsequently, the culture was centrifuged at 4000 × g for 15 min to remove bacteria. The supernatant was transferred to a sterile tube and stored at 4°C with a few drops of chloroform to ensure sterility. To determine the concentration of phage particles, the sterile supernatant was serially diluted 10 times; each dilution was added to a suspension of phage-susceptible bacteria (10^7^ bacteria in 3 ml of the melted top layer of 0.7% agar) and spread on agar plates. After overnight cultivation, plaques were counted and the resulting bacteriophage titer was expressed as plaque forming units (PFU)/ml.

In parallel, induction of phage production was tested using the same protocol with a slight modification, which included the addition of mitomycin C (final concentration 0.1 μg/ml) to the culture of lysogenic strains during the exponential growth phase (i.e., after 4 hr of cultivation at 37°C, 200 rpm); cultivation continued for an additional 3 hr. Phage production was determined using a plaque counting technique. A comparison of phage titers counted from cultures with and without addition of mitomycin C indicated inducibility of phage production.

### Activity of phages against clinically important *Salmonella* isolates

Using the identified phage producers, we examined the activity of phages against a set of 52 clinically important *S*. *enterica* subsp. e*nterica* isolates using the cross-test method described above. Clinical isolates belonged to common pathogenic serovars, i.e. Enteritidis (22 isolates), Typhimurium (13 isolates), Choleraesuis (4 isolates), Indiana (3 isolates), Derby (2 isolates), Mikawashima (2 isolates), Agona (1 isolate), Chester (1 isolate), Infantis (1 isolate), Java (1 isolate), Ohio (1 isolate), and San Diego (1 isolate). All these pathogenic strains were collected by The National Reference Laboratory for Salmonella, National Institute of Public Health (NIPH), Prague, Czech Republic, from patients living in the Czech Republic. The characteristics of the pathogenic strains are shown in S1 Table.

### Analysis of PCR amplification profiles of phages

DNA from all phages was isolated using a QIAGEN Lambda Midi Kit (Qiagen, Hilden, Germany) per the protocol recommended by the manufacturer. Total bacteriophage DNA was digested using *Eco*RI enzyme (New England Biolabs, Ipswich, USA), cloned into the pUC19 cloning vector (New England Biolabs); the resulting clones were sequenced. The resulting sequences were compared with the GenBank database using BLAST to identify similarities to other phages. Subsequently, 12 DNA regions representing different phage genes were selected for primer design. The DNA from all phages was used as a template for PCR amplification (Table 1) and up to 2 genetic regions originating from the same phage were used for further screening. A list of genetic loci and the corresponding primer sequences are shown in Table 1. Individual PCR reactions contained 0.4 μl of 10 mM dNTP mix, 1.8 μl of 10× ThermoPol Reaction buffer, 0.25 μl of each primer (100 pmol/μl), 0.05 μl of *Taq* polymerase (5000 U/ml), 1 μl of the tested phage DNA sample, and 15.2 μl of PCR grade water. PCR amplification was performed under the following cycling conditions: 94°C (2 min); 94°C (30 s), 60°C (30 s), 72°C (60 s), 30 cycles; 72°C (10 min). Resulting PCR products were sequenced with amplification primers. The resulting phage sequences were deposited in the GenBank under accession numbers KM202139, KM202140, KM202142 –KM202144, KM202151 –KM202154, KM202157, KM202158, KM202160, KM202164 –KM202167, KM202171 –KM202176, KM202179, KM202181 –KM202184, and KM202186 –KM202195.

**Table 1:** List of primers used for amplification and sequencing of several phage genome regions.

Analyzed genome region	Phage template for primer design	Primer name	Primer sequence (5'-3')	Amplicon length (bp)	Target region encoding
GA1	SEN22	F4c5-F	CAACTTGCGACTGCTCTTTG	474	Terminase small subunit
		F4c5-R	CGAAGGAAGCGTTAGACCTG		
GA2	SEN2	F3c7-F	GTTGGGTGGAAGAAGCTGAA	413	Terminase large subunit
		F3c7-R	AGCATCTGGCCCTGTATCTG		
GA3	SEN8	F20c3-F	TTCAGTGGTTGGTTCCATGA	471	Portal protein
		F20c3-R	GCGTTAAGAAGCCAGAAACG		
GA4	SEN8	F20c5-F	TTATTTGCTGCGCTGACATC	494	Scaffolding protein
		F20c5-R	TCAGAAGAAGGCCTGGCTAA		
GA5	SEN23	F6c1-F	GGCCGATCAGTTGCTTAAAA	232	Integrase
		F6c1-R	CTCATGCCCCAACATTTTCT		
GA6	SEN4	F9c2-F	GCCGTCTGATAACCCAGAAA	456	Methyltransferase
		F9c2-R	CGTTTTCCAGATCAGCCTGT		
GA7	SEN34	F38c2-F	AAGTGGGGAACTGCTGAAGA	487	Replication protein O
		F38c2-R	AACGCTGCCATAAGCTGACT		
GA8	SEN1	F1c3-F	AAAGGCCGGTTAGGTAGCTC	456	Replication protein
		F1c3-R	ATCGCTCGCATGTTTAACG		
GA9	SEN22	F4c4-F	AATGGATGCAGTCAGGGAAG	421	Nin locus
		F4c4-R	GTCAATCATCGCGTTTTCCT		
GA10	SEN5	F10c1-F	TTCGCAAATGAAATCGAGTG	443	Hypothetical protein
		F10c1-R	ATCGGCAACTTACCGTCATC		
GA11	SEN23	F6c4-F	GTTGTTCAGGCCGTTGATTT	284	Hypothetical protein
		F6c4-R	GTACATCGCCTGAAGGGAGA		
GA12	SEN34	F38c1-F	GTTTATGGCGCTGAAAAGGA	457	Hypothetical protein
		F38c1-R	GTTGTTCAGGCCGTTGATTT		

### Preparation of phage stock

Bacteriophage stock was prepared from a single selected plaque [22] that was resuspended in 100 μl of TY broth containing 0.01M CaCl_2_. This phage suspension was added to 0.7% TY agar containing 10^7^ phage-susceptible bacteria (i.e., they did not produce their own bacteriophages) and spread on agar plate. After overnight cultivation, the bacteriophage-containing top layer of TY agar, with confluent lysis, was collected into a sterile tube and resuspended in 3 ml of SM buffer (100 mM NaCl, 8 mM MgSO_4_*7H_2_O, 50 mM Tris-Cl, 0.01% (w/v) gelatine) and 0.5 ml of chloroform. The tube was incubated at 37°C for 20 min and the suspension was centrifuged at 4000 × g for 15 min to remove bacteria and agar residue. The supernatant, containing the phage particles, was transferred to a sterile tube and few drops of chloroform were added.

### Isolation of bacteriophage DNA for whole genome sequencing

The bacterial stocks originated from single plaques multiplied in indicator strains (i.e., did not produce their own phages) were used for propagation of phages and isolation of phage DNA (see above). Phages were propagated per previously published protocols [22]. Briefly, 200 ml of TY broth was inoculated with 2 ml of fresh phage indicator culture and cultivated for 4.5 hr (37°C, 250 rpm). Thereafter, 200 μl of 0.02M CaCl_2_ and phage particles (≈ 10^8^) from our phage stock were added and cultivation continued for an additional 4.5 hr (37°C, 250 rpm). Finally, the bacterial culture was lysed using chloroform (10 min, 37°C, 250 rpm). Bacterial nucleic acids in lysates were degraded using RNase A and DNase I (final conc. 1 μg/ml, 45 min, RT) and phage particles were precipitated using NaCl (1M, 1 hr, 4°C) and PEG 8000 (10%, 16 hr, 4°C). After centrifugation (11,000 × g, 11 min, 4°C), the pellet was resuspended in 5 ml of SM buffer and phage particles were purified using isopycnic ultracentrifugation in a CsCl gradient (87,000 × g, 2 hr, 4°C). The collected bacteriophage suspension was dialyzed with dialysis buffer (10 mM NaCl, 50 mM Tris-Cl, 10 mM MgCl_2_) for 2 ×1 hr. The dialyzed phage suspension was treated with EDTA (final conc. 20 mM), proteinase K (final conc. 50 μg/ml) and SDS (final conc. 0.5%) for 1 hr at 56°C. Phage DNA was isolated by phenol-chloroform, precipitated by isopropanol, and finally diluted in 50 μl of TE buffer.

### Whole-genome sequencing and analysis of phage genomes

Genomic DNA of 5 phages was sequenced using Ion Torrent™ next-gen sequencing technology (Life Technologies, Carlsbad, USA) at SeqOmics Biotechnology Ltd (Mórahalom, Hungary). All genomes were assembled *de novo* using SeqMan NGen software from a Lasergene v.10 genomics package (DNASTAR, Madison, USA). Genomes were annotated using RAST (Rapid Annotation using Subsystem Technology), a fully-automatic annotation service [23], followed by manual correction based on the BLASTX algorithm [24]. Genomes were deposited in GenBank under the following numbers: SEN1 (KT630644); SEN4 (KT630645), SEN5 (KT 630646), SEN22 (KT 630648), and SEN34 (KT 630649). Genomes of *Salmonella* phages were analyzed using the Lasergene program package (DNASTAR). BLAST comparisons of phage genomes were produced with Easyfig [25]. Average nucleotide identity (ANI) of phages was calculated by the JSspeciesWeb Server running MUMmer 3.0 software [26, 27].

### Electron microscopy

Phage samples for electron microscopy were prepared from phage stocks (originated from a single plaque) according to previously published protocols [22]. Phage suspensions were subsequently precipitated with polyethylene glycol (PEG) and purified using centrifugation in a CsCl gradient [22]. Bacteriophage particles were visualized using a negative staining method. Briefly, phage suspensions (5 μl) were placed on glow discharge activated carbon-coated grids [28]. After an adsorption period (30 s), the unabsorbed liquid material was removed using filter paper. The electron-microscopic grids were then stained with 10 μl of 1% phosphotungstic acid, 2% uranyl acetate, or 2% ammonium molybdate for 10–30 s; excess staining solution was removed using filter paper. Samples were viewed using a MORGAGNI 268D (FEI, Hillsboro, OR, USA) or a Philips CM 100 transmission electron microscope (FEI).

## Results

### Characterization of non-pathogenic *Salmonella enterica* strains and identification of lysogenic strains

All non-pathogenic *S*. *enterica* strains used in this study (n = 40) were classified into subspecies based on biochemical markers and serotyped using the White-Kaufmann-LeMinor scheme. A total of 16 strains belonged to *S*. *enterica* subsp. *salamae*, 5 strains to subsp. *arizonae*, 15 strains to subsp. *diarizonae*, and 4 strains to subsp. *houtenae*. Strains mostly came from human feces or other human material, and from waste water sludge. In 7 cases, the origin of the strain was unknown (S1 Table).

All forty *S*. *enterica* strains were tested against each other (i.e., in 1,600 individual tests), as potential phage producers and phage indicators. In addition to *S*. *enterica* strains, four standard *E*. *coli* phage-indicator strains (i.e., K12-Row, C6 (φ), B1, and P400) were used. The results are summarized in Table 2. Production of bacteriophages was observed in 15 non-pathogenic *S*. *enterica* strains (37.5%) (Sen1, Sen2, Sen4, Sen5, Sen6, Sen8, Sen14, Sen16, Sen22, Sen23, Sen24, Sen30, Sen31, Sen34, and Sen35) belonging to either subspecies *salamae* (8 strains) or *diarizonae* (7 strains). All phages formed small turbid plaques without a lytic halo. Plaque sizes varied from 0.3–1.6 mm in diameter. Bacteriophage titers obtained from non-induced cultures ranged from 1.3 × 10^3^ to 4.0 × 10^6^ PFU/ml. Moreover, mitomycin C induction of phages was effective in 8 out of 15 phage producers (Table 2), increasing the phage titer by two orders of magnitude.

**Table 2:** Phage producers and indicator strains of identified phages.

Phage producer (*S*. *enterica* subspecies)	Susceptible indicator strains		PFU/ml	Plaque diameter (mm)
	*S*. *enterica*	*E*. *coli*		
Sen1 (*salamae*)*	Sen4, Sen5, Sen6, Sen9, Sen11	P400, B1	2.8 × 10^4^	1.5 ± 0.06
Sen2 (*salamae*)*	Sen11, Sen17, Sen26	-	2.9 × 10^6^	0.8 ± 0.06
Sen4 (*salamae*)	Sen38	P400, B1	9.2 × 10^3^	0.5 ± 0.05
Sen5 (*salamae*)	Sen38	P400, B1	6.9 × 10^3^	0.5 ± 0.06
Sen6 (*salamae*)*	Sen9, Sen33	-	2.7 × 10^4^	0.3 ± 0.06
Sen8 (*salamae*)	Sen6, Sen9, Sen10, Sen36, Sen38, Sen40	-	1.5 × 10^5^	0.7 ± 0.05
Sen14 (*salamae*)*	Sen11	P400, B1	6.1 × 10^4^	0.5 ± 0.05
Sen16 (*salamae*)	Sen38, Sen6, Sen9, Sen10, Sen11, Sen40, Sen36	P400, B1	1.6 × 10^5^	1.6 ± 0.05
Sen22 (*diarizonae*)*	Sen26, Sen39, Sen34	-	4.0 × 10^6^	1.2 ± 0.08
Sen23 (*diarizonae*)*	Sen11, Sen22, Sen24, Sen39	-	3.4 × 10^4^	0.4 ± 0.07
Sen24 (*diarizonae*)	Sen26, Sen39	-	1.5 × 10^4^	1.1 ± 0.07
Sen30 (*diarizonae*)*	Sen9	-	7.2 × 10^3^	1.1 ± 0.05
Sen31 (*diarizonae*)	Sen9	-	1.3 × 10^3^	1.1 ± 0.06
Sen34 (*diarizonae*)*	Sen23, Sen24, Sen26, Sen39	-	5.2 × 10^4^	1.3 ± 0.07
Sen35 (*diarizonae*)	-	P400, B1	6.5 × 10^4^	0.7 ± 0.05

*phage production was induced by mitomycin C; PFU–plaque forming unit

Indicator strains used for preparation of phage stocks are underlined.

### Analysis of phage activity against pathogenic *S*. *enterica* clinical isolates

**Fig 1:**
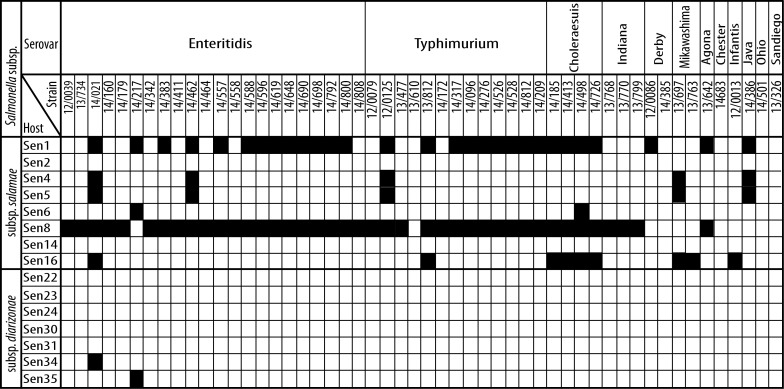
Activity of phages against pathogenic *Salmonella enterica* subsp. *enterica* isolates. Susceptibility of clinical isolates to phages is shown in black. “Host” refers to identified phage producers. Note the difference in the spectra of phages produced by strains from subsp. *salamae* and *diarizonae*.

Activity of all 15 phages from identified phage producers was tested against 52 pathogenic *S*. *enterica* subsp. *enterica* isolates belonging to common pathogenic serovars. The activity spectra of phages are shown in Fig 1. Two strains, i.e. Sen1 and Sen8, produced phages, which lysed the majority of pathogenic isolates, while phages produced by Sen4, Sen5, Sen6, Sen16, Sen34, and Sen35 strains inhibited less than 20% of isolates. Moreover, all pathogenic *Salmonella* isolates were resistant to phages produced by strains Sen2, Sen14, Sen22, Sen23, Sen24, Sen30, and Sen31. In general, phages from subsp. *salamae* showed broader activity against pathogenic salmonellae compared to phages from subsp. *diarizonae*.

### Analysis of phage PCR amplification profiles

**Fig 2:**
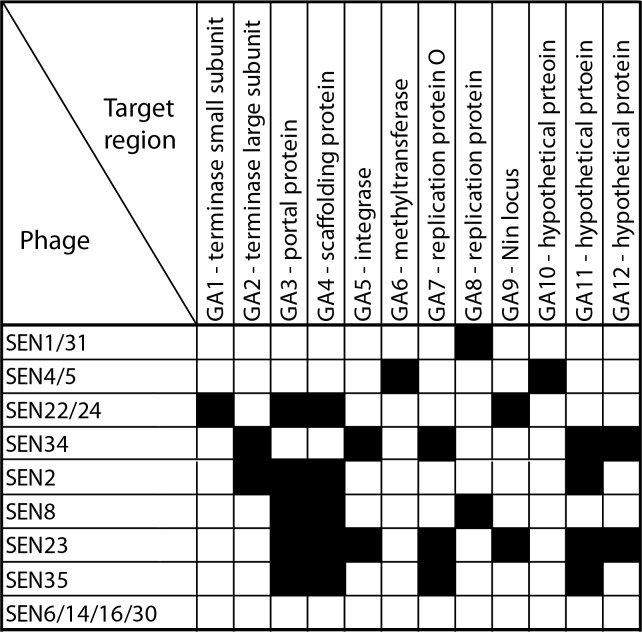
Phage PCR amplification profiles. Based on sequencing of cloned phage libraries, 12 phage DNA regions were selected for PCR amplification from all fifteen phages. Positive amplification results are shown in black.

Variability of identified phages (denoted as “SEN” throughout the manuscript) was analyzed using PCR amplification of several loci obtained during construction of small libraries of phages. Altogether, 12 short fragments (200–300 bp) from different genes were tested in all 15 phages (Fig 2). Phages SEN6, SEN14, SEN16, and SEN30 were negative for all PCR detected DNA regions. Three pairs of phages (i.e., SEN1 and SEN31, SEN4 and SEN5, and SEN22 and SEN24) had identical amplification profiles. The five remaining phages showed a unique spectra of PCR positive reactions. Altogether, nine different amplification profiles were identified. Finally, phages SEN1, SEN4, SEN5, SEN22, and SEN34 were selected for further analysis based on: i) variability in PCR profiles, ii) differences in the activity against pathogenic salmonellae, iii) classification of phage producers into different subspecies, and iv) quality of isolated DNA.

### Morphology of phage particles

**Fig 3:**
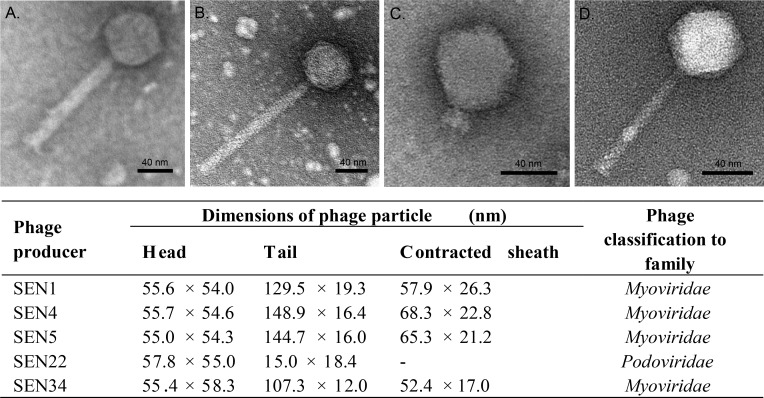
Morphology of *Salmonella* phages. Electron microscopy of negatively stained phage particles isolated from the producer strain Sen1 **(A)**, Sen4 and Sen5 (phage SEN4/5) **(B)**, Sen22 **(C),** and Sen34 **(D)**. While phage SEN22 belongs to family *Podoviridae*, other phages showed morphology of family *Myoviridae*. Bar scale represents 40 nm. Dimensions of phage virions are shown in table.

Phages SEN1, SEN4, and SEN5 from producers belonging to subsp. *salamae*, and phages SEN22 and SEN34 from producers belonging to subsp. *diarizonae* were characterized using electron microscopy. A phage suspension obtained from a single plaque was negatively stained and examined with a transmission electron microscope to determine phage morphology. The morphology of phage particles is summarized in Fig 3. Phages SEN1, SEN4, SEN5, and SEN34, shared the same morphology, i.e. a head with icosahedral symmetry and a long contractile tail. Diameters of icosahedral capsids ranged from 54.0–58.3 nm and the length of the tail, including the contractile sheath, ranged from 107.3–148.9 nm. In contrast, phage SEN22 had an icosahedral head (57.8 nm in width; 55 nm in length) with very short noncontractile tail (18.4 nm in width; 15.0 nm in length). Based on previous classifications [29], the first morphological group corresponds to the *Myoviridae* family, and the second morphological group corresponds to the *Podoviridae* family.

### Whole genome analysis

Whole genome sequencing of phages SEN1, SEN4, SEN5, SEN22, and SEN34 was performed. The main characteristics of phage genomes are shown in Table 3. Genome length of the sequenced phages ranged from 29.7–41.3 kb with G+C content ranging from 47.8–53.4%. Genome lengths were correlated with genome sizes, which were estimated from PFGE (data not shown), indicating that complete genome sequences had been obtained. While the *cos* site, identical to predicted cohesive ends of phage Psp3 (ggcgtggcggggaaagcat), was found for SEN1, sticky ends of phages SEN4 and SEN5 (ggcgaggcgggggaacgag) were more similar to the *cos* site of phage P2. In addition, a *pac* sequence (gaagatttatctgaagtcgtta) identical to that of P22 phage was identified for phage SEN22. Due to unusual SEN34 genome (see below), the packaging-responsible sequences were not identified in the SEN34 genome. All sequenced phages encoded integrase, a characteristic of temperate phages.

**Table 3:** Genome characteristics of newly sequenced *Salmonella* phages.

Phage	Host (*S*. *enterica* subsp.)	Genome size (bp)	No. of reads (average coverage)	G+C content (%)	No. of predicted genes	The most similar phage (e-value/ANI %)*	Genbank Acc. No.
**SEN1**	*salamae*	29733	9163 (67×)	53.01	43	Enterobacteria phage PsP3 (0.0/97.11%)	KT630644
**SEN4**	*salamae*	33509	14313 (94×)	53.36	47	no similarity^#^	KT630645
**SEN5**	*salamae*	33509	14619 (95×)	53.36	47	no similarity	KT630646
**SEN22**	*diarizonae*	41338	15702 (72×)	47.83	55	*Salmonella* phage epsilon34 (0.0/94.11%)	KT630648
**SEN34**	*diarizonae*	40740	19506 (108×)	49.91	63	no similarity	KT630649

*BLASTN analysis of whole genome phage sequences was performed against a database of viruses (taxid:10239) and results were ranked based on the total score. Average nucleotide identity (ANI) of the most similar phages was calculated by the JSspeciesWeb Server.

^#^phage genome sequences showing similarity in less than 10% of genome length were marked as “no similarity”

**Fig 4:**
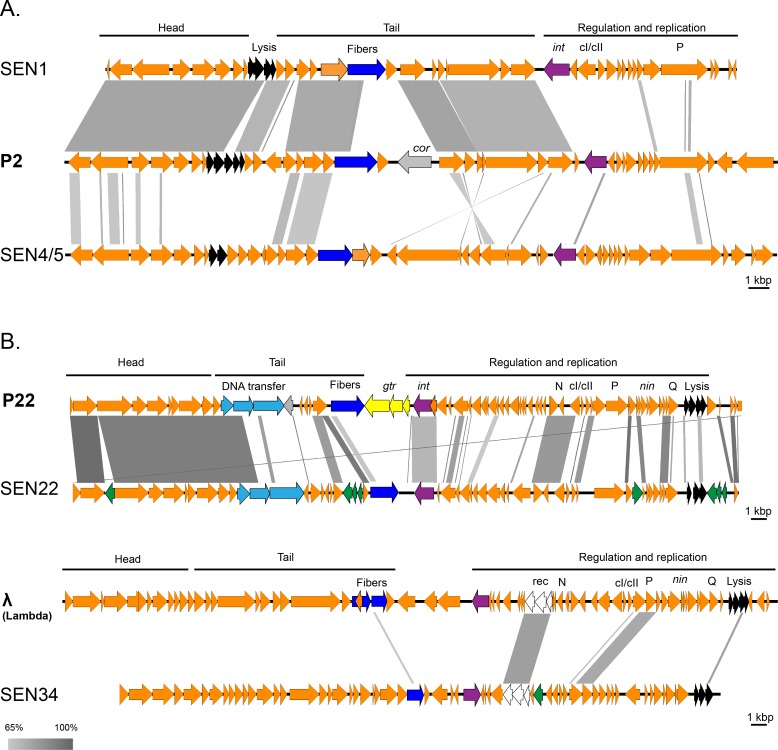
Genome analyses of sequenced phages SEN1, SEN4/5, SEN22, and SEN34. Genome comparison of phages isolated from subsp. *salamae* (A) and from subsp. *diarizonae* (B). Schematic organization of genes in phage genomes (head-tail-regulation-replication) is shown with bold black lines. The most variable genes (i.e., those encoding DNA transfer proteins and tail fibers) are shown in blue. Genes for integration (*int*) are violet, genes encoding lysis are black, the locus responsible for O antigen modification (*gtr*) is yellow, the locus for lysogenic conversion (*cor*) is grey, and the locus for recombination (*rec*) is white. Mobile elements (transposons, HNH endonucleases) are green and the remaining genes are orange. Comparisons of phage genomes were prepared using Easyfig software [25]; similarity between phages (> 65%) is shown using a gray scale (bottom left).

A BLASTN analysis of whole genomes (Table 3) revealed substantial similarity (ANI: 97.11%) between phage SEN1 and phage PsP3 (a phage belonging to the P2-like group) (GenBank No. AY135486). The genome of phage SEN1 was also very similar (ANI: 81.69%) to the prototype phage P2 (GenBank No. KC618326), especially in the morphogenesis region. Besides the genetic variability found in the region responsible for replication, the lysogenic conversion locus was not present in the SEN1 genome and the region encoding tail fibers differed between SEN1 and P2 phages (Fig 4A). In fact, the tail fiber gene of SEN1 (gp19) was related to another P2-like phage, *Salmonella* phage RE-2010 (GenBank No. HM770079).

Whole genome sequencing of phages SEN4 and SEN5 revealed that these phages were identical (Table 3), thus representing a single phage type, denoted as phage SEN4/5. Whole genome analysis of SEN4/5, based on BLASTN, found similarities to the prophage sequence within the genome of *Enterobacter cloacae* complex (GenBank No. CP012162), but not to any known phages (Table 3); however, BLASTP analysis of predicted SEN4/5 proteins showed a distant similarity to several P2-like phages (Fig 4A and S2 Table). Nonetheless, the average nucleotide identity with the P2 phage was calculated to be 0.00%. Thus, SEN4/5 appears to belong to the P2-like phages, but it is clearly different from previously known members.

Both SEN22 and SEN34 phages, isolated from subsp. *diarizonae*, showed larger genomes compared to subsp. *salamae* phages (Table 3). The genome of phage SEN22 was found to be related to several P22-like phages having the greatest similarity (ANI: 94.11%) to *Salmonella* phage ɛ34 (GenBank No. NC_011976). The SEN22 genome region encoding the head of the virion was more similar to prototype phage P22 than the region encoding the virion tail and the region responsible for regulation and replication (Fig 4B). In addition, four insertions of mobile elements were identified in the SEN22 genome, including a unique insertion of a homing endonuclease gene between the terminase gene and the portal protein gene.

BLASTN analysis of the SEN34 genome sequence revealed a low degree of similarity to the prophage sequence within the genome of *S*. *enterica* subsp. *enterica* serovar Newport str. CVM 21550 (GenBank No. CP010283), but not to any known phages (Table 3), while the BLASTP analysis of predicted proteins showed that there was a distant similarity between the SEN34 virion assembly genes and two uncharacterized phages including the *Burkholderia* phage Bups phi1 and *Acinetobacter* phage Ab105-1phi (S2 Table). Additionally, the region responsible for replication and regulation of SEN34 was similar to several Lambda-like phages (Fig 4B and S2 Table). When all these observations are taken together, SEN34 appears to be a new phage type (ANI with Lambda phage: 0.00%).

## Discussion

To date, almost 200 phage types have been identified in the genus *Salmonella* [30] and if prophages are included, the number is greater than 9,000 [18]. Prophages appear to be very common in *Salmonella*, as shown by production of 136 functional phages from 173 *S*. *enterica* subsp. *enterica* (serovar Typhimurium) isolates [31]. It has been calculated that there are, on average, 2.8 known prophages present per *Salmonella* genome [18]. While phages and prophages of subsp. *enterica* have been intensively studied (reviewed in [18, 30, 32]), information on phages in other *Salmonella* subspecies is scarce. In fact, putative prophage sequences were only recently identified in the whole genome sequences of two strains belonging to non-*enterica* subspecies, i.e. in subsp. *arizonae* (GenBank: CP000880.1; prophage Sari1; [33]), and subsp. *salamae* (GenBank: ATFA01000000; [34]). This study focused on phage production within strains *S*. *enterica* subspecies *salamae*, *arizonae*, *diarizonae*, and *houtenae*. While fifteen phage producers were identified in subspecies *salamae* and *diarizonae*, no producers were identified in subspecies *arizonae* and *houtenae*; however, it is unclear if this difference is due to the relatively low number of investigated strains in the second group (9 out of 40) or due to other factors.

Out of 15 identified phages, SEN1, SEN4/5, SEN22, and SEN34 were further characterized. Bacteriophages SEN1, SEN4/5, and SEN34 belonged to the *Myoviridae* family, while SEN22 showed morphology typical of the *Podoviridae* family. These findings are in accordance with the fact that the majority of known phages belongs to the *Myoviridae* family [35].

Analysis of phage genomes revealed that these phages represent novel types. All sequenced phages encoded integrase, a characteristic of temperate phages. While phages SEN1 and SEN22 showed close sequence relatedness to prototype phages P2 and P22, respectively, phages SEN4/5 and SEN34 were quite different from known phages. Our findings are in accordance with a study by Casjens and Grose [18], where SEN4/5 genomic sequences formed a new, well defined, subcluster within the P2-like cluster. Phage SEN34 was similar to several disparate Lambda-like phages, but did not belong to any of the 17 known lambda clusters described by Grose and Casjens [17]. Thus, phage SEN34 is a representative of a new lambda cluster. This is in accordance with a recent comprehensive study of enterobacterial prophages [18], where the authors analyzed the available phage genomes from GenBank database and defined a new lambda phage cluster (“temperate 26”) with prototype phage SEN34. Although phage SEN34 is genetically related to Lambda-like phages, morphologically it is a member of the *Myoviridae* family (in contrast to Lambda phages, which belong to the *Siphoviridae* family). A similar discrepancy between genomic relatedness and morphology classification has also been shown for the LP65 phage, which had a *Myoviridae* morphology, however, it had a genome organization similar to the Lambda phage [36].

The additional 10 unsequenced phages identified in this study, likely represent distinct phage types as revealed by (i) differences in amplification profiles of various genomic regions, (ii) analyses of the sources of *Salmonella* strains, (iii) the spectra of indicator strains, and (iv) inducibility with mitomycin C.

Genomes of temperate phages sequenced in our study showed genome mosaicism, where several parts of the phage genomes were related to several different phages. This is in agreement with previous comparative analyses showing that the morphogenesis region is relatively conserved, while genome mosaicism is prevalent in the early regions of phage genomes [17, 37–40].

Besides common mosaicism in the early region, genes encoding proteins interacting with the host showed increased genetic diversity. The DNA encoding receptor-binding tailspike protein domain is among the most highly exchanged parts of the tailed phages. It is evolutionarily useful for phages to acquire new receptor specificities by swapping this domain through horizontal gene transfer [33]. While sequenced phages produced by the subsp. *salamae* showed a similarity to the region encoding tail fibers in the P2-like *Salmonella* phage RE-2010, no sequence similarity was found for the SEN22 and SEN34 phages.

Phages produced by strains belonging to subsp. *salamae* showed a broader activity spectra against pathogenic isolates of subsp. *enterica* compared to phages from subsp. *diarizonae*. This is in accordance with results from a phylogenetic study of *Salmonella* subspecies [41], which showed a close relationship between subsp. *salamae* and subsp. *enterica*. The observed broad activity of phages SEN1 and SEN8 against pathogenic salmonellae opens up the possibility of finding therapeutic applications for these phages, however, the presence of lysogenic cycles in these phages makes such therapeutic applications rather potential.

## Conclusions

In this study, we determined phage production in *S*. *enterica* subspecies *salamae*, *arizonae*, *diarizonae*, and *houtenae*. Out of 15 identified phage producers, five complete phage genomes were determined and four different temperate phages were identified. Phages SEN1 and SEN4/5 clustered with P2-like phages, while phage SEN22 showed sequence relatedness to P22-like phages. Phage SEN34 was distantly related to Lambda-like phages (*Siphoviridae*), but had a morphology that was characteristic of *Myoviridae*.

## Supporting Information

S1 Table The list of *Salmonella* strains used in this study. (DOCX)

S2 Table BLASTP analysis of predicted proteins in sequenced phages. (DOCX)
