# Supplementary material for: Novel Temperate Phages of Salmonella enterica subsp. salamae and subsp. diarizonae and Their Activity against Pathogenic S. enterica subsp. enterica Isolates
Source: PLoS One. 2017 Jan 24;12(1):e0170734. doi: 10.1371/journal.pone.0170734 (PMC5261728; doi:10.1371/journal.pone.0170734)
Supplement: S1 Table — (DOCX) [file pone.0170734.s001.docx]

**The list of non-pathogenic *Salmonella* strains used in this study.**

| ***Salmonella* strain** | **Original strain identification from NIPH** | ***S. enterica* subspecies** | **Source** | **White-Kaufmann-LeMinor scheme** | | |
| --- | --- | --- | --- | --- | --- | --- |
|  |  |  |  | **Somatic (O) antigen** | **Flagellar (H) antigen** | |
|  |  |  |  |  | **Phase 1** | **Phase 2** |
| **Sen1** | 10/99 II | *salamae* | human origin | 6,7 | z_29_ | - |
| **Sen2** | 569/99 II | *salamae* | sludge | 47 | a | 1,5 |
| Sen3 | 3171/00 II | *salamae* | human feces | 42 | z_10_ | 1,2 |
| **Sen4** | 200/01 II | *salamae* | human feces | 48 | d | z_6_ |
| **Sen5** | 241/01 II | *salamae* | human feces | 48 | d | z_6_ |
| **Sen6** | 199/02 II | *salamae* | human feces | 43 | d | e,n,x,z_15_ |
| Sen7 | 172/03 II | *salamae* | human feces | 6,8 | z_29_ | e,n,x |
| **Sen8** | 244/03 II | *salamae* | human feces | 6,8 | l,z_28_ | z_6_ |
| Sen9 | 586/03 II | *salamae* | human feces | 6,7 | g,m,t | - |
| Sen10 | 252/04 II | *salamae* | human feces | 16 | z_4,_z_32_ | - |
| Sen11 | 261/04 II | *salamae* | human feces | 42 | z_10_ | 1,2 |
| Sen12 | 520/04 II | *salamae* | unknown | 30 | l,z_28_ | z_6_ |
| Sen13 | 13/05 II | *salamae* | unknown | 30 | l,z_28_ | z_6_ |
| **Sen14** | 311/96 II | *salamae* | sewage | nd | nd | nd |
| Sen15 | 13/98 II | *salamae* | human feces | 13,23 | z | 1,5 |
| **Sen16** | 77/05 II | *salamae* | unknown | 47 | a | 1,5 |
| Sen17 | 274/99 IIIa | *arizonae* | human origin | 53 | g,z_51_ | - |
| Sen18 | 125/01 IIIa | *arizonae* | sludge | 50 | k | z |
| Sen19 | 92/02 IIIa | *arizonae* | human feces | 41 | z_4_,z_23_ | - |
| Sen20 | 44/03 IIIa | *arizonae* | human feces | 48 | z_4_,z_24_ | - |
| Sen21 | 412/03 IIIa | *arizonae* | human feces | 41 | z_4_,z_23_ | - |
| **Sen22** | 89/00 IIIb | *diarizonae* | human origin | 48 | l,v | 1,5 |
| **Sen23** | 7/01 IIIb | *diarizonae* | unknown | 47 | l,v | 1,5 |
| **Sen24** | 257/01 IIIb | *diarizonae* | human feces | 50 | r | 1,5 |
| Sen25 | 23/02 IIIb | *diarizonae* | human feces | 38 | r | z |
| Sen26 | 38/02 IIIb | *diarizonae* | human throat | 50 | - | 1,5,7 |
| Sen27 | 350/03 IIIb | *diarizonae* | human feces | 48 | i | z |
| Sen28 | 422/03 IIIb | *diarizonae* | unknown | 38 | r | z |
| Sen29 | 539/03 IIIb | *diarizonae* | human feces | 48 | i | z |
| **Sen30** | 547/03 IIIb | *diarizonae* | human feces | 65 | z_10_ | e,n,x,z_15_ |
| **Sen31** | 33/04 IIIb | *diarizonae* | human feces | 65 | z_10_ | e,n,x,z_15_ |
| Sen32 | 454/04 IIIb | *diarizonae* | human feces | 50 | k | z |
| Sen33 | 17/05 IIIb | *diarizonae* | human feces | 60 | r | e,n,x,z_15_ |
| **Sen34** | 519/03 IIIb | *diarizonae* | human feces | 6,14 | l,v | z |
| **Sen35** | 18/98 IIIb | *diarizonae* | unknown | 16 | z_10_ | e,n,x,z_15_ |
| Sen36 | 192/98 IIIb | *diarizonae* | sludge | 47 | z_4_,z_23_ | - |
| Sen37 | 110/01 IV | *houtenae* | human feces | 38 | z_4_,z_23_ | - |
| Sen38 | 543/01 IV | *houtenae* | human feces | 16 | z_4_,z_23_ | - |
| Sen39 | 104/02 IV | *houtenae* | human feces | 48 | g,z_51_ | - |
| Sen40 | 337/04 IV | *houtenae* | unknown | 16 | z_4,_z_32_ | - |

nd – not determined; The phage producers are shown in **bold**.

**The list of pathogenic *Salmonella* strains used in this study**

| **Strain** | **Serovar** | **Sex** | **Birth** | **Source** | **Diagnosis** |
| --- | --- | --- | --- | --- | --- |
| 12/0013 | Infantis | M | 1946 | not specified | K57.2 (Diverticulosis of large intestine) |
| 12/0039 | Enteritidis | F | 1943 | hemoculture | R52.9 (Pain) |
| 12/0079 | Typhimurium | F | 1947 | hemoculture | A41.9 (Sepsis) |
| 12/0086 | Derby | M | 1941 | not specified | K57.3 (Diverticulosis of large intestine) |
| 12/0125 | Typhimurium | M | 1947 | hemoculture | I50.1 (Left ventricular failure) |
| 13/326 | San Diego | M | 2004 | hemoculture | R50.9 (Fever) |
| 13/477 | Typhimurium | M | 1939 | hemoculture | A09.0 (Infectious gastroenteritis and colitis) |
| 13/642 | Agona | F | 1928 | hemoculture | R50.9 (Fever) |
| 13/697 | Mikawashima | M | 1944 | hemoculture | I80.2 (Phlebitis and thrombophlebitis) |
| 13/734 | Enteritidis | F | 1937 | not specified | R17. (Jaundice) |
| 13/763 | Mikawashima | F | 1993 | feces | A09.0 (Infectious gastroenteritis and colitis) |
| 13/768 | Indiana | F | 1985 | feces | A09.0 (Infectious gastroenteritis and colitis) |
| 13/770 | Indiana | M | 1994 | feces | A09.0 (Infectious gastroenteritis and colitis) |
| 13/799 | Indiana | M | 2004 | feces | A09.0 (Infectious gastroenteritis and colitis) |
| 13/810 | Typhimurium | F | 1947 | feces | A02.0 (Salmonella enteritis) |
| 13/812 | Typhimurium | M | 1974 | feces | A09.0 (Infectious gastroenteritis and colitis) |
| 14/021 | Enteritidis | F | 1954 | hemoculture | A41.9 (Sepsis) |
| 14/096 | Typhimurium | M | 2013 | feces | A09.9 (Diarrhea) |
| 14/160 | Enteritidis | M | 1942 | hemoculture | G00.9 (Bacterial meningitis) |
| 14/172 | Typhimurium | F | 1925 | feces | A09.0 (Infectious gastroenteritis and colitis) |
| 14/179 | Enteritidis | F | 1949 | hemoculture | S30.0 (Contusion of lower back and pelvis) |
| 14/185 | Choleraesuis | M | 1955 | hemoculture | I50.9 (Heart failure) |
| 14/209 | Typhimurium | M | 1993 | feces | K52.9 (Noninfective gastroenteritis and colitis) |
| 14/217 | Enteritidis | M | 1960 | not specified | C71.7 (Malignant neoplasm of brain stem) |
| 14/276 | Typhimurium | M | 2000 | feces | E86. (Dehydration) |
| 14/317 | Typhimurium | M | 1987 | feces | A09.0 (Infectious gastroenteritis and colitis) |
| 14/342 | Enteritidis | M | 1949 | hemoculture | N17.8 (Acute kidney failure) |
| 14/383 | Enteritidis | F | 1958 | hemoculture | M05.9 (Rheumatoid arthritis) |
| 14/385 | Derby | M | 1934 | feces | A09.0 (Infectious gastroenteritis and colitis) |
| 14/386 | Java | F | 1995 | feces | A09.0 (Infectious gastroenteritis and colitis) |
| 14/411 | Enteritidis | M | 1944 | hemoculture | N30.0 (Acute cystitis with hematuria) |
| 14/413 | Choleraesuis | F | 1940 | hemoculture | R50.9 (Fever) |
| 14/462 | Enteritidis | M | 1923 | hemoculture | N30.0 (Acute cystitis with hematuria) |
| 14/464 | Enteritidis | M | 1957 | hemoculture | C09.1 (Malignant neoplasm of tonsillar pillar) |
| 14/498 | Choleraesuis | F | 1956 | hemoculture | I67.1 (Cerebral aneurysm, nonruptured) |
| 14/501 | Ohio | M | 1953 | feces | A09.0 (Infectious gastroenteritis and colitis) |
| 14/526 | Typhimurium | M | 1971 | hemoculture | N17.9 (Acute kidney failure) |
| 14/528 | Typhimurium | F | 1997 | feces | A09.9 (Infectious gastroenteritis and colitis) |
| 14/557 | Enteritidis | M | 1953 | not specified | K31.6 (Fistula of stomach and duodenum) |
| 14/558 | Enteritidis | M | 1949 | hemoculture | A49.9 (Bacterial infection) |
| 14/588 | Enteritidis | F | 1977 | hemoculture | C71.9 (Malignant neoplasm of brain) |
| 14/596 | Enteritidis | F | 1930 | hemoculture | R50.9 (Fever) |
| 14/619 | Enteritidis | M | 1942 | hemoculture | A02.0 (Salmonella enteritis) |
| 14/648 | Enteritidis | M | 1945 | not specified | I71.4 (Abdominal aortic aneurysm, without rupture) |
| 14/683 | Chester | M | 2013 | not specified | M13.9 (Arthritis) |
| 14/690 | Enteritidis | M | 1932 | hemoculture | J15.8 (Pneumonia) |
| 14/698 | Enteritidis | F | 1946 | hemoculture | A09.0 (Infectious gastroenteritis and colitis) |
| 14/726 | Choleraesuis | F | 1937 | hemoculture | R06.0 (Orthopnea) |
| 14/792 | Enteritidis | M | 1960 | hemoculture | E14.8 (Unspecified diabetes mellitus) |
| 14/800 | Enteritidis | F | 1953 | hemoculture | C82.9 (Follicular lymphoma) |
| 14/808 | Enteritidis | M | 1972 | hemoculture | A09.0 (Infectious gastroenteritis and colitis) |
| 14/812 | Typhimurium | M | 1993 | feces | A09.0 (Infectious gastroenteritis and colitis) |
