# Supplementary material for: Novel Temperate Phages of Salmonella enterica subsp. salamae and subsp. diarizonae and Their Activity against Pathogenic S. enterica subsp. enterica Isolates
Source: PLoS One. 2017 Jan 24;12(1):e0170734. doi: 10.1371/journal.pone.0170734 (PMC5261728; doi:10.1371/journal.pone.0170734)
Supplement: S2 Table — (DOCX) [file pone.0170734.s002.docx]

| **SEN4/5: BLASTP analysis of predicted proteins (Date of analysis: September 2016)** | | | | | | | |
| --- | --- | --- | --- | --- | --- | --- | --- |
| **Gene** | **Predicted protein** | **The most similar sequence [Phage]** | **Query cover** | **Identity** | **The second most similar sequence [Phage]** | **Query cover** | **Identity** |
| gene1 | portal vertex protein | P2 gpQ-like protein [Salmonella phage Fels-2] | 98% | 53% | capsid packaging protein [Salmonella phage RE-2010] | 90% | 56% |
| gene2 | terminase ATPase subunit | terminase ATPase subunit [Enterobacteria phage P88] | 98% | 56% | gp3 [Enterobacteria phage PsP3] | 97% | 48% |
| gene3 | capsid scaffolding protein | capsid scaffolding protein [Erwinia phage ENT90] | 91% | 47% | P2 gpO-like protein [Salmonella phage Fels-2] | 83% | 43% |
| gene4 | major capsid protein | Major head protein; Flags: Precursor [Serratia phage KSP20] | 98% | 56% | major capsid protein [Enterobacteria phage P88] | 98% | 54% |
| gene5 | terminase endonuclease subunit | terminase endonuclease subunit [Enterobacteria phage P88] | 96% | 45% |  |  |  |
| gene6 | head completion-stabilization protein | head completion protein [Serratia phage KSP20] | 98% | 52% | head completion-stabilization protein [Enterobacteria phage P88] | 86% | 50% |
| gene7 | tail component protein | tail component protein [Enterobacteria phage P88] | 98% | 52% | P2 gpX-like tail protein [Salmonella phage Fels-2] | 100% | 52% |
| gene8 | holin | holin [Enterobacteria phage P88] | 100% | 56% |  |  |  |
| gene9 | lysin | bacteriophage lysis protein; endolysin; lysozyme [Phage Gifsy-2] | 100% | 62% | putative lysozyme [Edwardsiella phage GF-2] | 100% | 59% |
| gene10 | hypothetical protein | hypothetical protein [Enterobacter phage Arya] | 97% | 45% |  |  |  |
| gene11 | tail protein | tail completion protein-like protein [Ralstonia phage RSA1] | 86% | 47% | gpR [Enterobacteria phage P2] | 87% | 41% |
| gene12 | tail completion protein | tail protein [Enterobacteria phage P88] | 96% | 43% |  |  |  |
| gene13 | hypothetical protein | hypothetical protein Acj61p098 [Acinetobacter phage Acj61] | 62% | 42% |  |  |  |
| gene14 | baseplate assembly protein V | baseplate assembly protein V [Escherichia phage pro483] | 99% | 59% | gp15 [Enterobacteria phage PsP3] | 98% | 59% |
| gene15 | baseplate assembly protein | baseplate assembly protein [Erwinia phage ENT90] | 92% | 52% | gp16 [Enterobacteria phage PsP3] | 95% | 49% |
| gene16 | baseplate assembly protein J | gp17 [Enterobacteria phage PsP3] | 99% | 69% | baseplate [Enterobacteria phage 186] | 99% | 70% |
| gene17 | Baseplate assembly protein I | P2 gpI-like baseplate assembly protein [Salmonella phage Fels-2] | 97% | 61% | same as RE2010 |  |  |
| gene18 | tail fiber protein | tail fiber protein [Salmonella phage RE-2010] | 92% | 88% | phage tail fiber protein [Enterobacteria phage P2] | 78% | 42% |
| gene19 | tail fiber protein | conserved hypothetical protein [Phage Gifsy-1] | 100% | 92% |  |  |  |
| gene20 | tail fibers protein | tail fiber assembly protein [Salmonella phage RE-2010] | 100% | 92% |  |  |  |
| gene21 | Phage tail protein | tail protein [Enterobacteria phage P88] | 99% | 53% | gp25 [Enterobacteria phage PsP3] | 62% | 56% |
| gene22 | tail length tape-measure protein | P2 gpT-like tail protein [Salmonella phage Fels-2] | 79% | 46% |  |  |  |
| gene23 | hypothetical protein | putative tail protein [Escherichia phage pro147] | 100% | 50% | gp23.5 [Enterobacteria phage PsP3] | 92% | 54% |
| gene24 | tail protein | gpE-like protein [Serratia phage KSP20] | 86% | 53% | gpE [Enterobacteria phage P2] | 80% | 43% |
| gene25 | major tail tube protein | P2 gpFII-like protein [Salmonella phage Fels-2] | 100% | 50% |  |  |  |
| gene26 | tail sheath monomer | hypothetical protein [Serratia phage KSP20] | 98% | 60% | tail sheath monomer [Enterobacteria phage P88] | 97% | 56% |
| gene27 | hypothetical protein | no similarity |  |  |  |  |  |
| gene28 | late control D protein | gene D protein [Enterobacteria phage P88] | 95% | 63% | gene D protein [Salmonella phage RE-2010] | 97% | 57% |
| gene29 | hypothetical protein | no similarity |  |  |  |  |  |
| gene30 | hypothetical protein | hypothetical protein [Acinetobacter phage Ab105-2phi] | 64% | 38% |  |  |  |
| gene31 | integrase | phage integrase [Enterobacteria phage P2] | 100% | 67% |  |  |  |
| gene32 | repressor protein C | gpC [Yersinia phage L-413C] | 82% | 59% | gpC [Enterobacteria phage P2] | 84% | 49% |
| gene33 | hypothetical protein | no similarity |  |  |  |  |  |
| gene34 | hypothetical protein | no similarity |  |  |  |  |  |
| gene35 | hypothetical protein | no similarity |  |  |  |  |  |
| gene36 | regulatory protein Cox | Cox [Yersinia phage L-413C] | 88% | 57% | Cox protein [Enterobacteria phage P2] | 89% | 49% |
| gene37 | hypothetical protein | no similarity |  |  |  |  |  |
| gene38 | hypothetical protein | hypothetical protein [Enterobacteria phage P88] | 80% | 42% |  |  |  |
| gene39 | hypothetical protein | no similarity |  |  |  |  |  |
| gene40 | hypothetical protein | no similarity |  |  |  |  |  |
| gene41 | methyl-directed repair DNA adenine methylase | putative DNA adenine methylase [Erwinia phage vB_EamM_Kwan] | 85% | 47% | DNA adenine methyltransferase [Salmonella phage RE-2010] | 84% | 42% |
| gene42 | DNA-cytosine methyltransferase | methyltransferase [Edwardsiella phage eiAU-183] | 100% | 56% | gp58 [Enterobacteria phage N15] | 100% | 48% |
| gene43 | replication protein | replication protein A [Cronobacter phage ESSI-2] | 70% | 47% | gp36 [Enterobacteria phage PsP3] | 72% | 47% |
| gene44 | hypothetical protein | no similarity |  |  |  |  |  |
| gene45 | DNA methylase | putative adenine-specific methyltransferase [Rhizobium phage vB_RleM_PPF1] | 60% | 46% |  |  |  |
| gene46 | hypothetical protein | no similarity |  |  |  |  |  |
| gene47 | hypothetical protein | hypothetical protein phiES15_001 [Cronobacter phage phiES15] | 91% | 24% |  |  |  |
|  |  |  |  |  |  |  |  |
|  |  | |  |  |  |  |  |

| **SEN34: BLASTP analysis of predicted proteins (Date of analysis: September 2016)** | | | | | | | |
| --- | --- | --- | --- | --- | --- | --- | --- |
| **Gene** | **Predicted protein** | **The most similar sequence [Phage]** | **Query cover** | **Identity** | **The second most similar sequence [Phage]** | **Query cover** | **Identity** |
| gene1 | terminase small subunit | terminase small subunit [Escherichia phage HK639] | 87% | 91% |  |  |  |
| gene2 | terminase large subunit | putative TerL [Burkholderia phage Bups phi1] | 99% | 63% |  |  |  |
| gene3 | portal protein | hypothetical protein [Acinetobacter phage Ab105-1phi] | 89% | 54% | putative portal protein [Burkholderia phage Bups phi1] | 91% | 52% |
| gene4 | head morphogenesis protein | hypothetical protein [Acinetobacter phage Ab105-1phi] | 97% | 47% | putative head morphogenesis protein [Burkholderia phage Bups phi1] | 96% | 44% |
| gene5 | head protein | hypothetical protein 2.44 [Burkholderia phage Bups phi1] | 96% | 44% |  |  |  |
| gene6 | hypothetical protein | hypothetical protein [Acinetobacter phage Ab105-1phi] | 99% | 43% |  |  |  |
| gene7 | major capsid protein | hypothetical protein [Acinetobacter phage Ab105-1phi] | 100% | 57% |  |  |  |
| gene8 | hypothetical protein | hypothetical protein [Acinetobacter phage Ab105-1phi] | 51% | 32% |  |  |  |
| gene9 | hypothetical protein | hypothetical protein [Acinetobacter phage Ab105-1phi] | 92% | 46% | hypothetical protein 5.3 [Burkholderia phage Bups phi1] | 93% | 41% |
| gene10 | hypothetical protein | hypothetical protein 5.4 [Burkholderia phage Bups phi1] | 92% | 42% | hypothetical protein [Acinetobacter phage Ab105-1phi] | 98% | 41% |
| gene11 | head-tail adaptor | hypothetical protein 5.6 [Burkholderia phage Bups phi1] | 95% | 42% |  |  |  |
| gene12 | hypothetical protein | hypothetical protein 5.7 [Burkholderia phage Bups phi1] | 97% | 37% |  |  |  |
| gene13 | hypothetical protein | hypothetical protein [Acinetobacter phage Ab105-1phi] | 97% | 48% |  |  |  |
| gene14 | hypothetical protein | hypothetical protein [Acinetobacter phage Ab105-1phi] | 100% | 49% |  |  |  |
| gene15 | hypothetical protein | hypothetical protein [Acinetobacter phage Ab105-1phi] | 92% | 28% |  |  |  |
| gene16 | tail tape measure protein | hypothetical protein [Acinetobacter phage Ab105-1phi] | 55% | 37% |  |  |  |
| gene17 | hypothetical protein | hypothetical protein [Acinetobacter phage Ab105-1phi] | 94% | 38% |  |  |  |
| gene18 | hypothetical protein | no similarity |  |  |  |  |  |
| gene19 | hypothetical protein | hypothetical protein 3.1 [Burkholderia phage Bups phi1] | 83% | 34% | hypothetical protein [Acinetobacter phage Ab105-1phi] | 96% | 28% |
| gene20 | hypothetical protein | ORF 1ab polyprotein [Beluga Whale coronavirus SW1] | 70% | 27% |  |  |  |
| gene21 | hypothetical protein | no similarity |  |  |  |  |  |
| gene22 | baseplate assembly protein | hypothetical protein 3.3 [Burkholderia phage Bups phi1] | 98% | 43% | hypothetical protein [Acinetobacter phage Ab105-1phi] | 99% | 38% |
| gene23 | hypothetical protein | putative bacteriophage protein [Enterobacteria phage CP-1639] | 100% | 60% |  |  |  |
| gene24 | hypothetical protein | putative oxidoreductase [Burkholderia phage Bups phi1] | 96% | 51% |  |  |  |
| gene25 | putative baseplate-related protein | hypothetical protein 3.9 [Burkholderia phage Bups phi1] | 97% | 49% | baseplate protein J |  |  |
| gene26 | putative tail component | hypothetical protein 3.10 [Burkholderia phage Bups phi1] | 98% | 39% |  |  |  |
| gene27 | tail fiber protein | gpS [Enterobacteria phage P1] | 55% | 42% | Various fragments… |  |  |
| gene28 | putative tail assembly chaperone | phage tail assembly chaperone [Edwardsiella phage eiAU] | 98% | 45% |  |  |  |
| gene29 | acyltransferase 3 | hypothetical protein [Escherichia phage TL-2011b] | 33% | 34% |  |  |  |
| gene30 | hypothetical protein | no similarity |  |  |  |  |  |
| gene31 | hypothetical protein | no similarity |  |  |  |  |  |
| gene32 | integrase | integrase [Enterobacteria phage HK544] | 100% | 61% |  |  |  |
| gene33 | hypothetical protein | transcriptional regulator AlpA family [Enterobacteria phage HK544] | 89% | 59% |  |  |  |
| gene34 | hypothetical protein | no similarity |  |  |  |  |  |
| gene35 | hypothetical protein | hypothetical protein STM2634.Gifsy1 [Phage Gifsy-1] | 98% | 95% |  |  |  |
| gene36 | methyl-directed repair DNA adenine methylase | Dam [Enterobacteria phage P7] | 93% | 51% |  |  |  |
| gene37 | exonuclease | phage exonuclease [Enterobacteria phage phi80] | 100% | 89% |  |  |  |
| gene38 | recombinational DNA repair protein RecT | bet [Enterobacteria phage lambda] | 100% | 95% |  |  |  |
| gene39 | host-nuclease inhibitor Gamma | host-nuclease inhibitor protein Gam [Enterobacteria phage Min27] | 98% | 72% |  |  |  |
| gene40 | penicillin-binding protein | Kil protein [Enterobacteria phage mEp237] | 98% | 81% |  |  |  |
| gene41 | homing endonuclease | homing endonuclease [Enterobacteria phage JenK1] | 91% | 35% |  |  |  |
| gene42 | putative DNA-binding protein | hypothetical protein mEp237_038 [Enterobacteria phage mEp237] | 96% | 57% |  |  |  |
| gene43 | putative DNA-binding protein | prophage repressor [Enterobacteria phage mEp237] | 91% | 78% |  |  |  |
| gene44 | regulatory protein | prophage anti-repressor [Enterobacteria phage mEp237] | 98% | 51% |  |  |  |
| gene45 | putative DNA-binding protein | bacteriophage transcriptional activator; Lambda gpCII analog [Phage Gifsy-2] | 100% | 100% | putative CII [Salmonella phage SPN3UB] | 100% | 99% |
| gene46 | replication protein O | DNA replication protein [Enterobacteria phage lambda] | 100% | 58% |  |  |  |
| gene47 | replication protein P | DNA replication protein [Enterobacteria phage lambda] | 98% | 81% |  |  |  |
| gene48 | hypothetical protein | hypothetical protein SP016_00285 [Salmonella phage FSL SP-016] | 89% | 98% |  |  |  |
| gene49 | hypothetical protein | conserved protein of unknown function [Escherichia phage RCS47] | 42% | 54% |  |  |  |
| gene50 | hypothetical protein | no similarity |  |  |  |  |  |
| gene51 | DNA polymerase III theta subunit | Hot [Enterobacteria phage P1] | 80% | 69% |  |  |  |
| gene52 | hypothetical protein | no similarity |  |  |  |  |  |
| gene53 | hypothetical protein | hypothetical protein [Stx2-converting phage Stx2a_F422] | 99% | 49% |  |  |  |
| gene54 | DNA-damage-inducible protein I | hypothetical protein mEp237_048 [Enterobacteria phage mEp237] | 100% | 75% | DinI [Salmonella phage SPN3UB] | 100% | 66% |
| gene55 | hypothetical protein | hypothetical protein mEp237_049 [Enterobacteria phage mEp237] | 100% | 94% |  |  |  |
| gene56 | hypothetical protein | hypothetical protein SPN3UB_0060 [Salmonella phage SPN3UB] | 100% | 99% | hypothetical protein STM1020.Gifsy2 [Phage Gifsy-2] | 100% | 99% |
| gene57 | hypothetical protein | hypothetical protein STM1021.1n.Gifsy2 [Phage Gifsy-2] | 100% | 100% | hypothetical protein mEp237_051 [Enterobacteria phage mEp237] | 100% | 61% |
| gene58 | NinG | NinG [Phage Gifsy-1] | 100% | 99% |  |  |  |
| gene59 | hypothetical protein | hypothetical protein STM1022.1n.Gifsy2 [Phage Gifsy-2] | 100% | 98% |  |  |  |
| gene60 | putative antitermination protein | prophage antitermination protein; late gene regulator; gpQ [Phage Gifsy-2] | 100% | 99% |  |  |  |
| gene61 | holin | bacteriophage lysis protein; holin [Phage Gifsy-2] | 100% | 100% |  |  |  |
| gene62 | lyisin | bacteriophage lysis protein; endolysin; lysozyme [Phage Gifsy-2] | 100% | 97% |  |  |  |
| gene63 | endopeptidase, outer membrane lytic protein Rz | bacteriophage lysis protein; Rz [Phage Gifsy-1] | 95% | 91% |  |  |  |

| **SEN1: BLASTP analysis of predicted proteins (Date of analysis: December 2016)** | | | | | | | |
| --- | --- | --- | --- | --- | --- | --- | --- |
| **Gene** | **Predicted protein** | **The most similar sequence [Phage]** | **Query cover** | **Identity** | **The second most similar sequence [Phage]** | **Query cover** | **Identity** |
| gene1 | hypothetical protein | no similarity |  |  |  |  |  |
| gene2 | portal protein | portal vertex protein [Salmonella phage FSL SP-004] | 100% | 99% | gp1 [Salmonella virus PsP3] | 100% | 95% |
| gene3 | terminase ATPase subunit | gp3 [Salmonella virus PsP3] | 100% | 99% |  |  |  |
| gene4 | capsid scaffolding protein | capsid-scaffolding protein [Salmonella phage FSL SP-004] | 100% | 99% | gp4 [Salmonella virus PsP3] | 100% | 99% |
| gene5 | major capsid protein | capsid protein [Salmonella phage FSL SP-004] | 100% | 99% | gp5 [Salmonella virus PsP3] | 100% | 94% |
| gene6 | terminase endonuclease subunit | R protein [Escherichia virus 186] | 100% | 96% | gp6 [Salmonella virus PsP3] | 100% | 96% |
| gene7 | head completion-stabilization protein | phage head completion-stabilization protein [Escherichia virus P2] | 100% | 67% |  |  |  |
| gene8 | tail completion protein | phage tail protein [Salmonella phage FSL SP-004] | 100% | 99% | gp8 [Salmonella virus PsP3] | 100% | 97% |
| gene9 | holin | holin [Salmonella phage FSL SP-004] | 100% | 99% | gp9 [Salmonella virus PsP3] | 100% | 98% |
| gene10 | 1,4-beta-N-acetylmuramidase | lysin [Salmonella phage FSL SP-004] | 100% | 99% | gp10 [Salmonella virus PsP3] | 100% | 97% |
| gene11 | lysis regulatory protein LysB | LysB [Salmonella phage FSL SP-004] | 100% | 98% | gp11 [Salmonella virus PsP3] | 100% | 91% |
| gene12 | lysis protein LysC | LysC [Salmonella phage FSL SP-004] | 100% | 100% | gp12 [Salmonella virus PsP3] | 100% | 98% |
| gene13 | tail protein | gp13 [Salmonella virus PsP3] | 100% | 100% |  |  |  |
| gene14 | tail completion protein | gp14 [Salmonella virus PsP3] | 100% | 99% | hypothetical protein SP004_00065 [Salmonella phage FSL SP-004] | 100% | 98% |
| gene15 | baseplate assembly protein V | gp15 [Salmonella virus PsP3] | 100% | 99% | phage baseplate assembly protein [Salmonella phage FSL SP-004] | 100% | 99% |
| gene16 | baseplate assembly protein W | gp16 [Salmonella virus PsP3] | 100% | 99% |  |  |  |
| gene17 | baseplate assembly protein | gp17 [Salmonella virus PsP3] | 100% | 98% | baseplate assembly protein [Salmonella phage FSL SP-004] | 100% | 98% |
| gene18 | tail fibers protein | gp18 [Salmonella virus PsP3] | 100% | 99% | phage tail protein [Salmonella phage FSL SP-004] | 100% | 98% |
| gene19 | putative tail fibers protein | tail fiber protein [Salmonella phage RE-2010] | 100% | 65% |  |  |  |
| gene20 | tail fibers protein | tail fiber assembly protein [Salmonella phage RE-2010] | 99% | 82% |  |  |  |
| gene21 | tail sheath monomer | gp21 [Salmonella virus PsP3] | 100% | 98% | major tail sheath protein [Salmonella phage FSL SP-004] | 100% | 94% |
| gene22 | major tail tube protein | gp22 [Salmonella virus PsP3] | 100% | 100% |  |  |  |
| gene23 | tail protein | gp23 [Salmonella virus PsP3] | 100% | 99% |  |  |  |
| gene24 | putative tail protein | gp23.5 [Salmonella virus PsP3] | 100% | 97% |  |  |  |
| gene25 | tail tape measure protein | gp24 [Salmonella virus PsP3] | 100% | 99% | phage tail tape measure protein [Salmonella phage FSL SP-004] | 99% | 90% |
| gene26 | tail protein | F protein [Escherichia virus 186] | 99% | 94% | hypothetical protein SP004_00120 [Salmonella phage FSL SP-004] | 99% | 94% |
| gene27 | late control D protein | gp26 [Salmonella virus PsP3] | 100% | 99% | tail protein [Salmonella phage FSL SP-004] | 99% | 94% |
| gene28 | integrase | gp27 [Salmonella virus PsP3] | 100% | 100% |  |  |  |
| gene29 | hypothetical protein | gp28 [Salmonella virus PsP3] | 100% | 100% |  |  |  |
| gene30 | Bacteriophage CI repressor | CI [Salmonella virus PsP3] | 100% | 99% |  |  |  |
| gene31 | hypothetical protein | gp30 [Salmonella virus PsP3] | 95% | 78% |  |  |  |
| gene32 | regulatory protein CII | CII [Escherichia virus 186] | 100% | 98% | gp31 [Salmonella virus PsP3] | 100% | 80% |
| gene33 | hypothetical protein | Fil [Escherichia virus 186] | 100% | 92% | gp32 [Salmonella virus PsP3] | 66% | 86% |
| gene34 | hypothetical protein | Dhr [Escherichia virus 186] | 100% | 92% |  |  |  |
| gene35 | hypothetical protein | gp33 [Salmonella virus PsP3] | 88% | 85% |  |  |  |
| gene36 | putative zinc-finger containing protein | gp34 [Salmonella virus PsP3] | 98% | 96% |  |  |  |
| gene37 | hypothetical protein | Orf83 [Escherichia virus 186] | 95% | 74% |  |  |  |
| gene38 | hypothetical protein | hypothetical protein HK106_029 [Enterobacteria phage HK106] | 93% | 39% |  |  |  |
| gene39 | replication protein | gp36 [Salmonella virus PsP3] | 95% | 96% |  |  |  |
| gene40 | hypothetical protein | TumA [Salmonella virus PsP3] | 100% | 100% |  |  |  |
| gene41 | DNA-damage-inducible protein DinI | TumB [Salmonella virus PsP3] | 100% | 97% |  |  |  |
| gene42 | hypothetical protein | no similarity |  |  |  |  |  |
| gene43 | hypothetical protein | no similarity |  |  |  |  |  |

| **SEN22: BLASTP analysis of predicted proteins (Date of analysis: December 2016)** | | | | | | | |
| --- | --- | --- | --- | --- | --- | --- | --- |
| **Gene** | **Predicted protein** | **The most similar sequence [Phage]** | **Query cover** | **Identity** | **The second most similar sequence [Phage]** | **Query cover** | **Identity** |
| gene1 | terminase small subunit | terminase small subunit [Salmonella virus P22] | 100% | 100% | hypothetical protein SP25_21 [Salmonella phage 25] | 100% | 100% |
| gene2 | terminase large subunit | terminase large subunit [Salmonella virus P22] | 100% | 99% |  |  |  |
| gene3 | homing endonuclease | endonuclease of the HNH family with predicted DNA-binding module in the C-terminus [Xanthomonas phage Xp10] | 85% | 39% |  |  |  |
| gene4 | portal protein | Gp1 [Salmonella enterica bacteriophage SE1] | 100% | 97% | portal protein [Salmonella phage vB_SemP_Emek] | 100% | 97% |
| gene5 | apsid and scaffolding protein | scaffolding protein [Salmonella virus P22] | 100% | 99% |  |  |  |
| gene6 | capsid protein | coat protein [Enterobacteria phage ST64T] | 100% | 99% |  |  |  |
| gene7 | hypothetical protein | hypothetical protein SPN9CC_0053 [Salmonella phage SPN9CC] | 100% | 94% | hypothetical protein P22gp06 [Salmonella virus P22] | 100% | 91% |
| gene8 | DNA stabilization protein | DNA stabilization protein [Salmonella phage SPN9CC] | 100% | 100% | head completion protein [Salmonella phage vB_SemP_Emek] | 100% | 99% |
| gene9 | DNA stabilization protein | packaged DNA stabilization protein [Salmonella phage SPN9CC] | 100% | 97% | Gp10 [Salmonella phage ST160] | 100% | 97% |
| gene10 | DNA stabilization protein | Gp26 [Salmonella enterica bacteriophage SE1] | 100% | 93% | putative structural protein [Salmonella phage vB_SemP_Emek] | 100% | 91% |
| gene11 | capsid and scaffolding protein | virion stability protein [Salmonella phage vB_SemP_Emek] | 100% | 99% | virion stability factor [Salmonella virus P22] | 100% | 98% |
| gene12 | DNA transfer protein | Gp7 [Salmonella enterica bacteriophage SE1] | 100% | 97% |  |  |  |
| gene13 | DNA transfer protein | hypothetical protein SP22_74 [Salmonella phage 22] | 47% | 95% | Gp20 [Salmonella phage ST160] | 47% | 95% |
| gene14 | DNA transfer protein | Ejection protein [Salmonella phage epsilon34] | 95% | 95% |  |  |  |
| gene15 | hypothetical protein | hypothetical protein epsilon34_gp14 [Salmonella phage epsilon34] | 100% | 95% |  |  |  |
| gene16 | hypothetical protein | no similarity |  |  |  |  |  |
| gene17 | hypothetical protein | no similarity |  |  |  |  |  |
| gene18 | regulatory protein | phage regulatory protein [Enterobacteria phage CUS-3] | 96% | 85% |  |  |  |
| gene19 | hypothetical protein | no similarity |  |  |  |  |  |
| gene20 | putative antirepressor protein | Ant [Salmonella virus P22] | 100% | 84% |  |  |  |
| gene21 | misc feature - mobile element |  |  |  |  |  |  |
| gene22 | putative antirepressor protein | antirepressor [Salmonella phage vB_SemP_Emek] | 100% | 95% | antirepressor [Salmonella phage vB_SemP_Emek] |  |  |
| gene23 | tail fibers protein | Tailspike protein [Salmonella phage epsilon34] | 26% | 64% |  |  |  |
| gene24 | integrase | Tyrosine integrase [Salmonella phage epsilon34] | 98% | 87% |  |  |  |
| gene25 | formate dehydrogenase N alpha subunit | no similarity |  |  |  |  |  |
| gene26 | EaA protein | EaA protein [Salmonella phage vB_SosS_Oslo] | 100% | 55% | EaA [Salmonella virus P22] | 98% | 47% |
| gene27 | hypothetical protein | no similarity |  |  |  |  |  |
| gene28 | hypothetical protein | Eae/HNH endonuclease fusion protein [Salmonella phage c341] | 21% | 100% |  |  |  |
| gene29 | hypothetical protein | hypothetical protein epsilon34_gp32 [Salmonella phage epsilon34] | 100% | 100% |  |  |  |
| gene30 | hypothetical protein | hypothetical protein mEpX1_032 [Enterobacteria phage mEpX1] | 100% | 91% |  |  |  |
| gene31 | hypothetical protein | hypothetical protein SEA_LUCKY10_50 [Gordonia phage Lucky10] | 83% | 43% |  |  |  |
| gene32 | exodeoxyribonuclease VIII | exonuclease [Vibrio phage pYD38-B] | 97% | 34% |  |  |  |
| gene33 | hypothetical protein | hypothetical protein mEpX1_035 [Enterobacteria phage mEpX1] | 100% | 94% |  |  |  |
| gene34 | hypothetical protein | hypothetical protein SPN9CC_0014 [Salmonella phage SPN9CC] | 100% | 96% |  |  |  |
| gene35 | c-type lectin precursor | hypothetical protein [Pseudomonas phage phi2] | 99% | 57% |  |  |  |
| gene36 | superinfection exclusion | SieB [Salmonella enterica bacteriophage SE1] | 100% | 97% |  |  |  |
| gene37 | antitermination protein N | 24 [Enterobacteria phage ST104] | 100% | 97% |  |  |  |
| gene38 | putative repressor protein | repressor protein [Salmonella phage ST64T] | 100% | 99% |  |  |  |
| gene39 | putative repressor protein | antirepressor protein [Salmonella phage ST64T] | 100% | 100% |  |  |  |
| gene40 | cII protein | transcriptional activator [Salmonella phage ST64T] | 100% | 100% |  |  |  |
| gene41 | hypothetical protein | hypothetical protein P22gp50 [Salmonella virus P22] | 100% | 94% |  |  |  |
| gene42 | replication protein O | DNA replication protein O [Enterobacteria phage mEp043 c-1] | 100% | 99% |  |  |  |
| gene43 | DNA primase/helicase | DNA replication protein [Salmonella phage epsilon34] | 100% | 99% |  |  |  |
| gene44 | NinB DNA recombination | hypothetical protein Stx2-86_gp71 [Stx2-converting phage 86] | 100% | 84% |  |  |  |
| gene45 | HNH homing endonuclease | hypothetical protein Stx2-86_gp72 [Stx2-converting phage 86] | 100% | 78% |  |  |  |
| gene46 | NinD protein | NinD [Salmonella phage epsilon34] | 82% | 98% |  |  |  |
| gene47 | NinF protein | NinF [Salmonella phage vB_SemP_Emek] | 98% | 91% |  |  |  |
| gene48 | hypothetical protein | hypothetical protein [Cronobacter phage ENT47670] | 100% | 96% |  |  |  |
| gene49 | endodeoxyribonuclease rusA | endodeoxyribonuclease [Salmonella phage ST64T] | 100% | 96% |  |  |  |
| gene50 | NinH protein | NinH [Salmonella virus P22] | 100% | 100% |  |  |  |
| gene51 | NinZ protein | gp72 [Enterobacteria phage ES18] | 100% | 97% |  |  |  |
| gene52 | antitermination protein Q | gp73 [Enterobacteria phage ES18] | 100% | 95% |  |  |  |
| gene53 | class II holin | gp13 [Enterobacteria phage ST104] | 100% | 100% |  |  |  |
| gene54 | lysin | lysin [Salmonella phage vB_SemP_Emek] | 100% | 96% |  |  |  |
| gene55 | outer membrane lytic protein Rz | endopeptidase [Salmonella phage SPN9CC] | 92% | 95% |  |  |  |
| gene56 | misc feature - mobile element |  |  |  |  |  |  |
| gene57 | hypothetical protein | hypothetical protein epsilon34_gp69 [Salmonella phage epsilon34] | 100% | 98% |  |  |  |
